# Gastric Duplication Cyst Presenting as Acute Abdomen: A Case Report

**Published:** 2010-08-14

**Authors:** Kanchan Kayastha, Afzal Sheikh

**Affiliations:** Department of Pediatric Surgery, The Children's Hospital and the Institute of Child Health Lahore, Pakistan

**Keywords:** Gastric duplication cyst, Acute abdomen, Peritonitis

## Abstract

Gastric duplication cysts are rare variety of gastrointestinal duplications. Sometimes they may present with complications like hemorrhage, infection, perforation, volvulus, intussusception and rarely neoplastic changes in the gastric duplication cyst. We present one and half year old male child who developed sudden abdominal distension with pain and fever for two days. Ultrasound revealed a cystic mass in the hypochondrium and epigastric regions. On exploration an infected and perforated gastric duplication cyst was found. Surgical excision of most part of cyst wall with mucosal stripping of the rest was performed. Histopathology confirmed the diagnosis of gastric duplication cyst. Early surgical intervention can result in good outcome.

## INTRODUCTION

Gastric duplication cysts constitute about 2-7 % of all gastrointestinal duplications. Majority of gastric duplication cysts are large and non-communicating. Most of the cases of gastric duplication cysts present in early age, however, in some cases patient may remain asymptomatic for a long period and might present with sudden onset of abdominal distension, pain, signs of obstruction, peritonitis etc. [1]. In this report we present a patient with complicated gastric duplication cyst.

## CASE REPORT

A male baby of one and half year presented in emergency with fever, abdominal pain and distension for two days. Fever was high grade and not associated with rigors and chills. Pain abdomen was continuous and patient had with few bouts of non bilious, non projectile vomiting. The past medical and surgical history was unremarkable. General physical examination revealed temp 101o F, pulse 100/min and respiratory rate 30/min. On abdominal examination generalized rigidity and guarding were present. No mass or viscera could be palpated. Bowel sounds were audible. A clinical diagnosis of acute peritonitis was made. X-ray abdomen was unremarkable and ultrasound showed a cystic structure of 4cm x 5cm size in the left hypochondrium and epigastrium. Urgent laparotomy was planned after initial stabilization.

At operation a cyst having intimate contact with the greater curvature of the stomach was found (Fig. 1). It was perforated at its posterior surface and about 500ml of pussy fluid was drained from the peritoneal cavity. The cyst was not communicating with the stomach but shared a common wall. Excision of major part of cyst was done with mucosal stripping of the residual wall attached to the stomach. Peritoneal lavage was done and a drain placed. Post-operative recovery was uneventful. Patient was discharged on 8th day following surgery. Histopathology revealed gastric mucosa and smooth muscles in the wall of cyst. At six months follow up patient remained well. 

**Figure:**
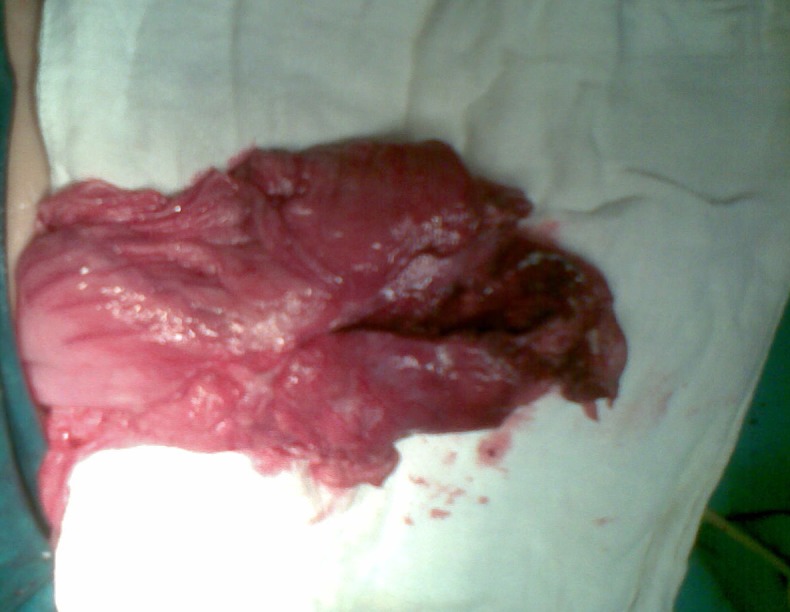
Figure 1: Showing gastric duplication cyst (surgically opened) attached intimately at greater curvature of stomach

## DISCUSSION

Gastric duplications are very uncommon congenital anomalies. They usually arise at greater curvature of stomach. Almost all gastric duplications are cystic in nature. Usually they are non-communicating. In our case gastric duplication was at greater curvature, cystic in nature and non-communicating. The clinical presentations of gastric duplication cysts depends upon site, size, communication with part of the alimentary tract and associated complications [2 , 3].

In complicated cases patient may present with an acute abdomen, peritonitis or even pancreatitis. Other complications include hemorrhage, infection, perforation of the cyst and compression on surrounding structures. Clinical features thus vary. Our patient presented with fever, pain and abdominal distension suggestive of complications like infection and perforation of the cyst [4 , 5].

The differential diagnoses of gastric duplication cyst include omental cyst, mesenteric cyst, choledochal cyst, ovarian cyst, hydronephrosis etc. Ultrasound abdomen, contrast GI studies, CT-scan and MRI are helpful diagnostic modalities. In our case ultrasound abdomen did show a cyst [1 , 5].

The treatment of gastric duplication cyst is surgical resection of the cyst. Due to its close connection with the adjacent gut usually it is very difficult to completely excise therefore partial resection with the mucosal stripping of the remaining cyst is recommended as done in present case. The diagnosis of duplication cyst is based upon presence of GIT mucosa in cyst with smooth muscle coat in the wall. Cyst must have an intimate contact with any part of GIT. In our patient all these features were present. Early diagnosis and prompt surgical intervention with optimal surgical procedure carries a good prognosis [1 , 3 , 6].

## Footnotes

**Source of Support:** Nil

**Conflict of Interest:** None declared
